# Supplementary material for: Neutrophil to lymphocyte ratio as an early indicator for ureteral catheterization in patients with renal colic due to upper urinary tract lithiasis
Source: PLoS One. 2022 Jun 29;17(6):e0270706. doi: 10.1371/journal.pone.0270706 (PMC9242459; doi:10.1371/journal.pone.0270706)
Supplement: Attachment — Submitted filename: Respone to Reviewers.docx [file pone.0270706.s001.docx]

PONE-D-21-23297

Neutrophil to lymphocyte ratio as an early indicator for ureteral catheterization in patients with renal colic due to upper urinary tract lithiasis

**Reviewer #1:** The study seems interesting and up-to-date. If the authors can clarify several issues as outlined below, the article can be more impressive.

Comment #1

- In the article, the NLR cut-off value was determined as 3.5 with reference to a previously published article. In the cited study, the NLR mean value was found to be 1.65 and the upper limit value was 3.53. Also, in their study authors stated that "NLR above 2.1 was associated with an increase of the probability for DJS insertion". Would the results have been different if the NLR cut-off value had been taken as 2.1?

Response

We agreed with the reviewer and re-analyzed our data taking NLR cut-off value of 2.1 and excluding patients with hematological diseases according to Reviewer’s #2 request. The new results did not change the major conclusions. The new data were changed accordingly throughout the whole manuscript including Tables and Figures.

Comment #2

- In the study, it was emphasized that NLR was a predictor for upper urinary tract decompression. In the method, the indications for emergency decompression of the upper urinary system were described. How many patients underwent upper urinary system decompression at the first admission and how many in the following period?

Response
Decompression of upper urinary tract was performed at the first admission in all indicated patients. This was clarified under the section of “Patients and Methods”

Comment #3

- In Table 2, "Death within a Month" rate in the high NLR group is remarkable. Did these deaths occur due to complications related to upper urinary tract stones or due to other reasons?

Response

The rate of death mentioned in in the results and Table 2 relates to overall mortality.

**Reviewer #2:** I would like to congratulate the study about a current issue. The importance of using neutrophil to lymphocyte ratio which is a novel diagnostic biomarker for disease such as kidney stone is increasing recently. I hope that such studies will contribute to the literature. However, I think this study which had a large number of included patients and including more than ten years can be published after correcting some minor points.

Comment #1
-The linguistics of the article should be revised by a native English speaker. Also the article should be checked to correct the spelling errors.

Response
The manuscript was re-edited by a native English speaker and rechecked for typos.

Comment #2
-The incidence and prevalence of Incidence of acute renal colic/nephrolithiasis presenting to the emergency department should also be mentioned in the manuscript.

Response
Between 2015 and 2019, a total of 828,000 patients were admitted to our emergency department making the incidence of acute renal colic 1.9% of all patients referring to our emergency department with a prevalence of 106 per 100,000 persons of the whole population in our Southern district. This info was added to Results accordingly.

Comment #3
-Hematological disease or leukemia should be added to the exclusion criteria.
-Also specific contributions by the authors individually part should be added to the manuscript.

Response
Following the reviewer’s request, we excluded patients with hematological diseased in our re-analysis. The new data were changed accordingly throughout the whole manuscript including Tables and Figures.

The specific contribution of all authors was added to manuscript.

Comment #4
- According to the my opinion, in the new trials, it may be possible to produce more valuable studies by adding information about stone characterization such as stone localization, number, Hounsfield Unit and type of kidney stone.

Response
We fully agree with reviewer and future studies will include the aforementioned data. This was emphasized in the limitations of the study in the original manuscript.
