# Supplementary material for: Neutrophil to lymphocyte ratio as an early indicator for ureteral catheterization in patients with renal colic due to upper urinary tract lithiasis
Source: PLoS One. 2022 Jun 29;17(6):e0270706. doi: 10.1371/journal.pone.0270706 (PMC9242459; doi:10.1371/journal.pone.0270706)
Supplement: Attachment — Submitted filename: Review 2.docx [file pone.0270706.s002.docx]

I thank the authors for preparing this original article. In general, the authors took into account and made the necessary revisions in the article. Only one thing catches my attention in the manuscript. There is probably a numerical mistake in the sentence "Fifty-seven patients (4.1%) underwent early DJS insertion" in the Results of the Abstract section. After the correction of this mistake, it would be more appropriate to accept the article. After the correction of this mistake, the manuscript could be acceptable for publication.
