# Supplementary material for: Neutrophil to lymphocyte ratio as an early indicator for ureteral catheterization in patients with renal colic due to upper urinary tract lithiasis
Source: PLoS One. 2022 Jun 29;17(6):e0270706. doi: 10.1371/journal.pone.0270706 (PMC9242459; doi:10.1371/journal.pone.0270706)
Supplement: Attachment — Submitted filename: Respone to Reviewers.2.docx [file pone.0270706.s003.docx]

PONE-D-21-23297R1

Neutrophil to lymphocyte ratio as an early indicator for ureteral catheterization in patients with renal colic due to upper urinary tract lithiasis

**Reviewer #1:** 

Comment #1

Only one thing catches my attention in the manuscript. There is probably a numerical mistake in the sentence "Fifty-seven patients (4.1%) underwent early DJS insertion" in the Results of the Abstract section.

Response

We thank the reviewer for drawing our attention for the numerical mistake appearing in the abstract: writing 57 instead of 557. The mistake was corrected accordingly.
